# *TCP24* modulates secondary cell wall thickening and anther endothecium development

**DOI:** 10.3389/fpls.2015.00436

**Published:** 2015-06-24

**Authors:** Han Wang, Yanfei Mao, Jun Yang, Yuke He

**Affiliations:** National Key Laboratory of Plant Molecular Genetics, Shanghai Institute of Plant Physiology and Ecology, Shanghai Institutes for Biological Sciences, Chinese Academy of SciencesShanghai, China

**Keywords:** *Arabidopsis*, *TCP24*, male sterility, anther dehiscence, secondary wall thickening, SRDX

## Abstract

miR319-targeted *TCP* genes are believed to regulate cell division in leaves and floral organs. However, it remains unknown whether these genes are involved in cell wall development. Here, we report that *TCP24* negatively regulates secondary wall thickening in floral organs and roots. The overexpression of the miR319a-resistant version of *TCP24* in *Arabidopsis* disrupted the thickening of secondary cell walls in the anther endothecium, leading to male sterility because of arrested anther dehiscence and pollen release. Several genes linked to secondary cell wall biogenesis and thickening were down-regulated in these transgenic plants. By contrast, the inhibition of TCP24 using the ectopic expression of a TCP24-SRDX repressor fusion protein, or the silencing of *TCP* genes by miR319a overexpression, increased cell wall lignification and the enhanced secondary cell wall thickening. Our results suggest that *TCP24* acts as an important regulator of secondary cell wall thickening and modulates anther endothecium development.

## Introduction

Anther dehiscence is a multistage process that involves coordinated programmed events in specific cells, including degeneration of the middle layer and the tapetum, thickening of the endothecium, degradation of septum cells, and breakage of stomium cells (Goldberg et al., 1993; Sanders et al., 1999; Wilson et al., 2011). Secondary wall thickening of the endothecium generates the tensile force necessary to rupture the stomium and in turn, release the pollen grains (Keijzer, 1987; Bonner and Dickinson, 1989). The importance of this process has been demonstrated by genetic analysis. A loss-of-function mutation of *MYB26* disrupts secondary thickening of the anther walls, resulting in non-dehiscent anthers (Dawson et al., 1999; Steiner-Lange et al., 2003; Yang et al., 2007). Two NAC domain transcription factors, *NST1* and *NST2*, function redundantly in regulating endothecium wall thickening and act downstream of *MYB26.* Overexpression of these two genes results in ectopic secondary thickening in various tissues (Mitsuda et al., 2005). Mutations in *IRREGULAR XYLEM* (*IRX*) and receptor-like protein kinase 2 (*RPK2*) genes also lead to the defective secondary wall thickening of the anther (Brown et al., 2005; Mizuno et al., 2007; Hao et al., 2014). Other genes, such as *CA2* (carbonic anhydrase 2), *AHP4* (*Arabidopsis* histidine-containing phosphotransfer factor 4), *SAF1* (secondary wall thickening-associated F-box 1) and *CBSX2* (cystathionine β-synthase domain-containing protein), negatively regulate this process, and the overexpression of these genes in *Arabidopsis* leads to anther non-dehiscent phenotypes (Jung et al., 2008, 2013; Villarreal et al., 2009; Kim et al., 2012).

The *TEOSINTE BRANCHED1*, *CYCLOIDEA*, and *PCF* (*TCP*) family encodes plant-specific transcription factors, which contain a conserved bHLH motif that allows DNA binding and protein–protein interactions (Cubas et al., 1999; Martin-Trillo and Cubas, 2010). The TCP members are grouped into two classes based on sequence homology: class I and class II *TCPs* (Cubas et al., 1999). It is believed that class I *TCP* genes promote cell division, while class II genes act antagonistically to inhibit cell division (Li et al., 2005). Duplication and diversification events over millions of years have generated a large family of 24 *TCP* genes in *Arabidopsis* of which 11 belong to the class II subfamily (Martin-Trillo and Cubas, 2010). A functional analysis shows that the class II *TCP* genes regulate several aspects of plant development. *Arabidopsis BRANCHED1* (*BRC1*) and *BRC2*, both closely related to the *TEOSINTE BRANCHED1* from maize (Doebley et al., 1997), are involved in suppressing axillary bud outgrowth (Aguilar-Martinez et al., 2007). *TCP2*, *TCP3*, *TCP4*, *TCP10*, and *TCP24* are the targets of miR319a/JAW. The down-regulation of these genes by overexpression of miR319a in *jaw-D* mutants generates larger leaves with crinkled surfaces owing to the extended cell proliferation along leaf margins (Palatnik et al., 2003). Conversely, hyper-activation of *TCP4* results in decreased cell proliferation, resulting in smaller leaves (Sarvepalli and Nath, 2011). miR319a-targeted genes function redundantly with *TCP5*, *TCP13*, and *TCP17* to coordinate the maintenance of undifferentiated fates in the shoot apical meristem and the promotion of the differentiated status in leaves (Koyama et al., 2007; Efroni et al., 2008). This coordination is achieved via the negative regulation of *CUP*-*SHAPED COTYLEDON* (*CUC*) genes, and TCP3 can directly activate the expression of *miR164A*, *ASYMMETRIC LEAVES1*, *INDOLE-3-ACETIC ACID3/SHORT HYPOCOTYL2 (IAA3/SHY2)*, and *At1g29460* to suppress *CUC* expression (Koyama et al., 2010). miR319a-targeted TCPs can interact with ASYMMETRIC LEAVES2 and repress the expression of *BREVIPEDICELLUS* and *KNAT2* genes by binding to their promoters causing normal leaf development (Li et al., 2012b). It is reported that a transcriptional repressor, TIE1, recruits co-repressors TOPLESS/TOPLESS-related proteins to repress the activities of class II *TCP* genes (Tao et al., 2013).

Previous studies showed that these *CINCINNATA* (*CIN*)-like *TCP* genes were expressed differentially in various organs, indicating that they might play important roles in many aspects of plant development (Koyama et al., 2007). In this study, we used a reverse genetic approach to investigate the function of *TCP24*. Overexpression of *TCP24* led to non-dehiscent anthers owing to the lack of secondary wall thickening in the endothecium, while fusing it with an EAR motif repressor domain (SRDX) caused enhanced lignin deposition in the anther endothecium, as well as other tissues, suggesting that *TCP24* functions as a negative regulator of secondary wall thickening.

## Materials and Methods

### Plant Materials and Growth Conditions

The wild type and transgenic plants of *Arabidopsis thaliana* used in this study were of Columbia ecotype (Col-0). Seeds were surface sterilized in 70% ethanol for 1 min, followed by 0.1% HgCl_2_ for 10 min, then washed five times in sterile distilled water, and plated on solid 1% sugar Murashige and Skoog medium. The plates were sealed with parafilm, incubated at 4°C in the dark for 2 days, and then moved to a growth room at 22°C with 16 h light. Two weeks later, the seedlings were transplanted carefully to peat soil in plastic pots, moved to a growth chamber in the phytotron of Institute of Plant Physiology and Ecology, and grown at 22°C with 16 h of light per day.

### Gene Cloning and Transformation

The full length CDS of *TCP24* was amplified from cDNA. *mTCP24* was generated by site-directed mutagenesis method using the QuikChange^®^ Lightning Site-Directed Mutagenesis Kit (Stratagene Catalog #210518) with appropriate primers. To construct the plasmid *p35S:mTCP24*, vector PJP100 (*p35S:mTCP2*, obtained from Dr. Weigel’s lab) was modified by replacing *mTCP2* with *mTCP24*. The 35S promoter of *p35S:mTCP24* was replaced with the *TCP24* promoter (2.7 kb fragment upstream from the translational start site) for the construction of *pTCP24:mTCP24*. *p35S:TCP24SRDX* was generated in our modified pCAMBIA3301 binary vector by fusing *TCP24* with the EAR motif repressor domain SRDX under the control of the 35S promoter. The plasmids were introduced into *Agrobacterium tumefaciens* strain GV3101 for plant transformation using the floral dip method as described previously (Li et al., 2012a). The transgenic plants of *p35S:mTCP24* and *pTCP24:mTCP24* were selected on plates containing 1/2 Murashige and Skoog media supplemented with 50 mg/L kanamycin, while *p35S:TCP24SRDX* plants were selected using 40 mg/L phosphinothricin.

### *In Situ* Hybridization

The full-length coding sequence of *TCP24* was polymerase chain reaction (PCR) amplified and cloned into pBluescript SK. Digoxigenin-labeled sense and antisense probes were synthesized with T7 or T3 RNA polymerase (Roche). Inflorescences from wild type and transgenic plants were pretreated and hybridized as described previously (Liu et al., 2011). Locked nucleic acid (LNA)-modified probe of miR319a was synthesized and labeled with DIG at the 3′ end and used for *in situ* hybridization.

### Real-Time PCR

The total RNA of inflorescences (with opened flowers removed) was extracted using TRIzol Reagent (Invitrogen) and treated with DNase I (TaKaRa) to remove DNA contamination. For cDNA synthesis, ∼4 μg RNA was reverse-transcribed using PrimeScript^®^ Reverse Transcriptase (TaKaRa) with oligo(dT) primers according to the manufacturer’s protocol. A quantitative real-time PCR analysis was performed using the Rotor-Gene 3000 system (Corbett Research, Mortlake, NSW, Australia) using SYBR Premix Ex Taq (Takara). *ACTIN* mRNA was used as an internal control, and the comparative threshold cycle (2^-ΔΔCt^) method was used to determine relative transcript levels. Three biological replicates and three technical replicates were performed. The gene specific primers for reverse-transcription PCR were shown in Supplementary Table S1.

### Histology

Inflorescences of 5- to 6-week-old wild type and transgenic plants were fixed in formalin/acetic acid/alcohol (FAA) and embedded in paraffin (Sigma). Then, 7 μm sections were stained with 0.05% (w/v) toluidine blue (Sigma) at 37°C for 15 min and then washed with water. For the analysis of semi-thin sections, samples fixed in FAA were embedded in epoxy resin. Then, 2-μm-thick sections were cut with glass knives, affixed to glass slides, and stained in 0.05% (w/v) toluidine blue. The sections were observed under a light microscope (Olympus model BX 51).

To visualize lignin deposition, plant tissues were stained with phloroglucinol-HCl solution (1.25 g of phloroglucinol dissolved in 25 ml of 95% ethanol and 10 ml of concentrated HCl), and observed under a dissecting microscope. To examine the secondary wall thickening in the endothecium, anthers were placed onto glass slides with clearing fluid. The solution was prepared from lactic acid, chloral hydrate, phenol, clove oil and xylene in the ratio 2:2:2:2:1, respectively, by weight (Herr, 1971). The anthers were observed by microscopy using differential interference contrast optics.

## Results

### Overexpression of *TCP24* Disrupts Anther Dehiscence in *Arabidopsis*

**FIGURE 1:**
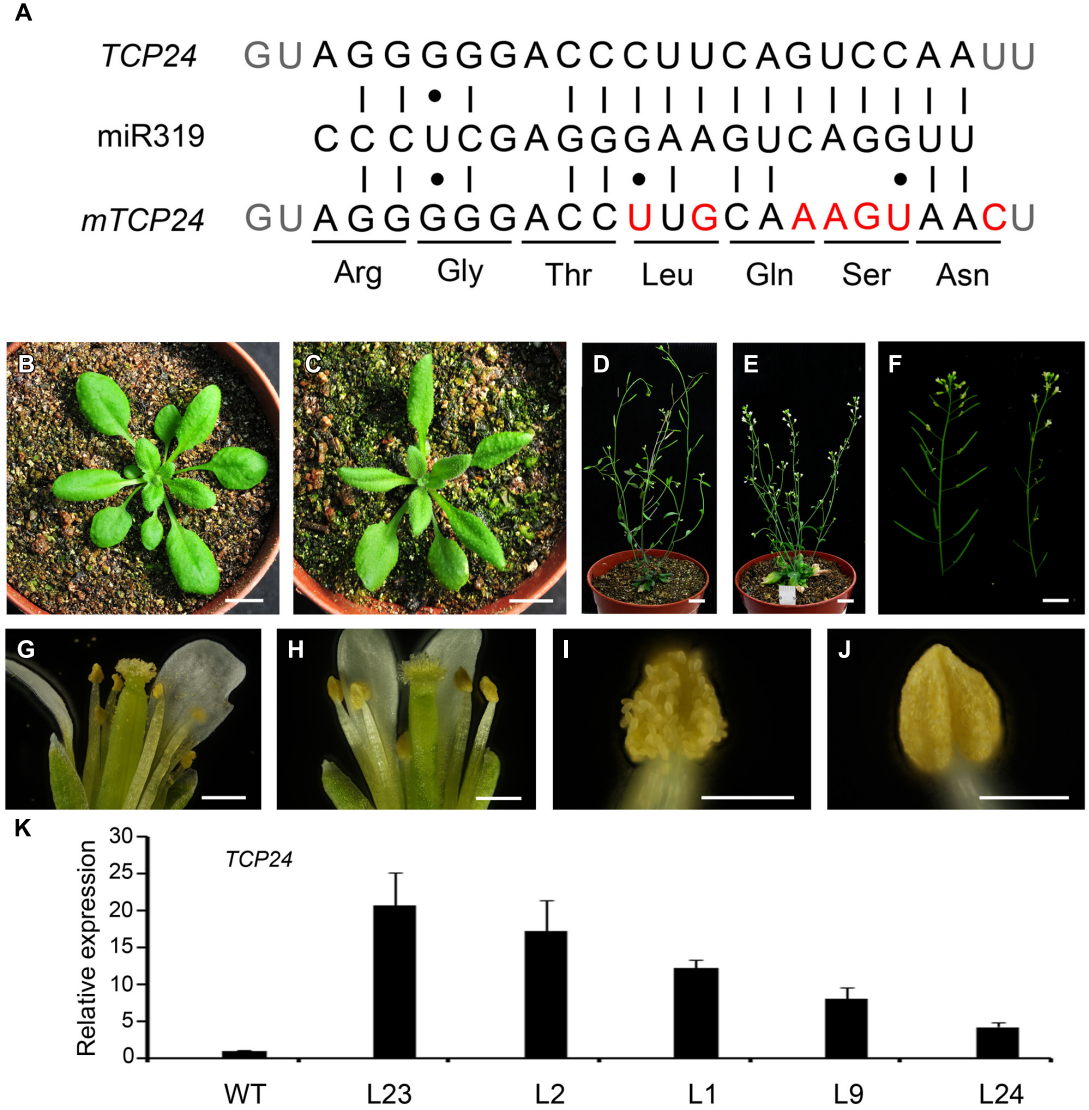
**Phenotypes of *p35S:mTCP24* plants. (A)** Diagram of *p35S:mTCP24* construct. **(B,C)** Plants of the wild type **(B)** and *p35S:mTCP24* line (L23; **C**) at the rosette stage. **(D,E)** Plants of the wild type **(D)** and L23 **(E)** at the inflorescence stage. **(F)** Inflorescences of the wild type (left) and L23 plants (right). **(G,H)** Open flowers showing the four whorls of floral organs in the wild type **(G)** and L23 **(H)**. **(I,J)** Anthers of the wild type **(I)** and L23 **(J)**. **(K)** Real-time PCR analysis of *TCP24* expression in the transgenic lines. Scale bars: 1 cm in **(B–F)**; 500 μm in **(G,H)**; 200 μm in **(I,J)**.

Mutations in single *CIN*-like *TCP* genes do not generate visible phenotypes owing to the redundancy among these genes (Koyama et al., 2007). To characterize the role of *TCP24* during plant development, we first constructed *mTCP24*, the miR319a-resistant version of *TCP24*, which contains nucleotide substitutions in the miR319a-binding region that do not change the encoded amino acid sequence (**Figure 1A**), as they did for *mTCP2*, *mTCP3*, and *mTCP4* (Palatnik et al., 2003; Koyama et al., 2007). In the *p35S:mTCP24* plants, the rosette leaves were turned slightly downward (**Figures 1B,C**), the number of branches increased compared with the wild type (**Figures 1D,E**), and importantly, the flowers were partially or completely sterile (**Figure 1F**). Under optical microscopy, anther dehiscence was arrested (**Figures 1G–J**), albeit to different extents between the transgenic lines. Transcripts of *TCP24* were elevated in these independent transgenic lines compared with wild type (**Figure 1K**). Among these transgenic lines, the higher the *TCP24* expression was, the higher the male sterility was, indicating a correlation between the expression levels of *TCP24* and the severity of the sterile phenotypes (Supplementary Figure S1). The L2 and L23, two completely sterile lines, set seeds when they were pollinated with the wild type pollen, indicating that *p35S*:*mTCP24* did not affect female fertility.

### *TCP24* Suppresses Secondary Wall Thickening of the Anther Endothecium

**FIGURE 2:**
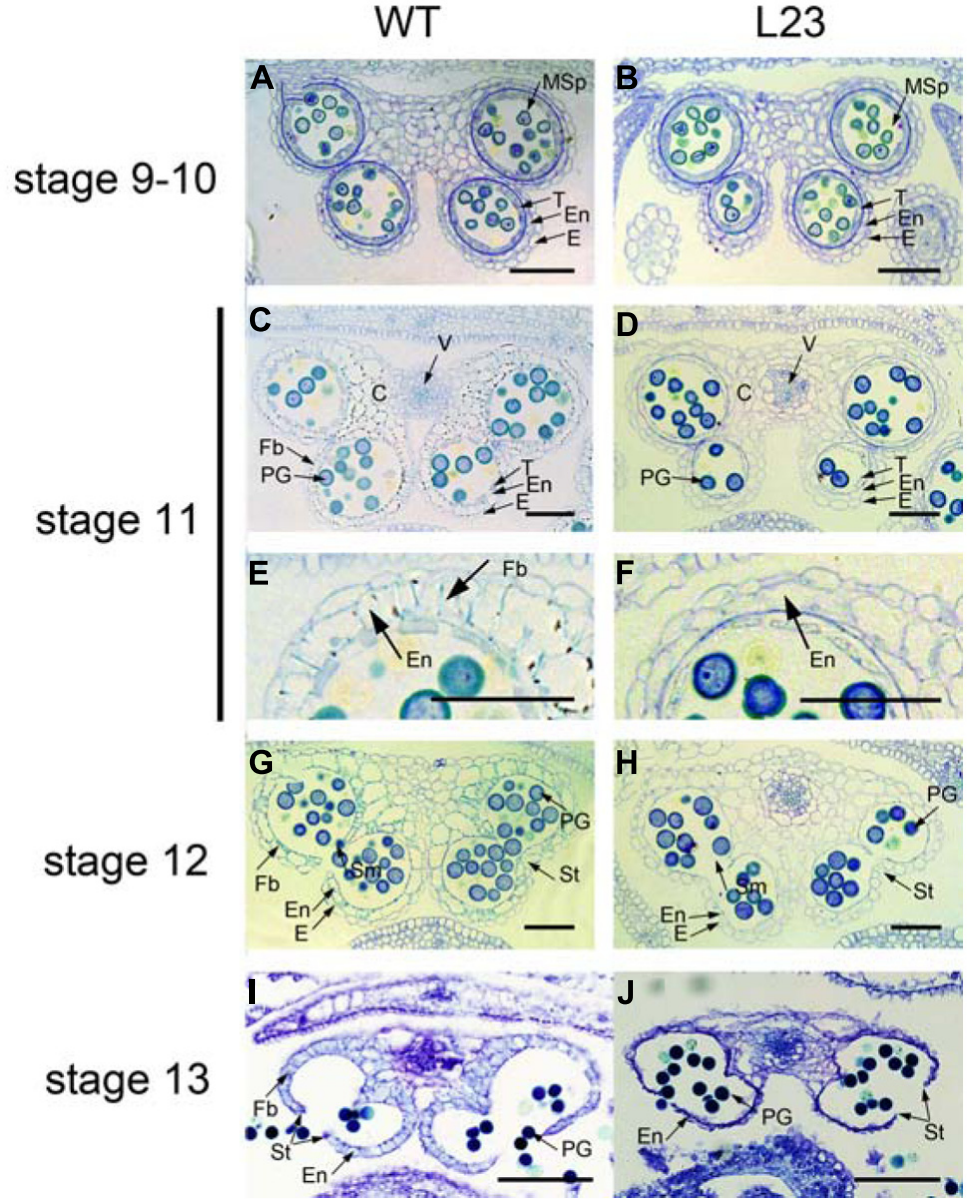
**Defects of *p35S:mTCP24* anthers in secondary cell wall thickening. (A,B)** Cross sections of the wild type **(A)** and L23 **(B)** anthers at stage 9. **(C,D)** Cross sections of the wild type **(C)** and L23 **(D)** anthers at stage 11. **(E,F)** Close-up of cross sections of the wild type **(E)** and L23 **(F). (G,H)** Cross sections of the wild type **(G)** and L23 **(H)** anthers at stage 12. **(I,J)** Cross sections of the wild type **(I)** and L23 **(J)** anthers at stage 13. Anther stages were defined according to Sanders et al. (1999). C, connective; E, epidermis; En, endothecium; Fb, fibrous bands; Msp, microspore; PG, pollen grain; Sm, septum; St, stomium; T, tapetum; V, vascular region. Scale bars: 50 μm in **(A–H)**; 100 μm in **(I,J)**.

Anther dehiscence requires the degeneration of some tissues and subsequent differentiation of other tissues, including epidermis, stomium, endothecium, and septum (Goldberg et al., 1993). To verify the defects in anther dehiscence in *p35S:mTCP24* plants, we examined the anther endothecium of line L23. During stages 9–10 (Sanders et al., 1999) when microspores were formed, L23 anthers were indistinguishable frm those of the wild type (**Figures 2A,B**). At stage 11, the tapetum of the wild type anthers was degenerated and the endothecium thickened, forming bands of striated spring-like structures (**Figures 2C,E**). However, secondary thickening in L23 anthers was not observed, as fibrous bands were absent in the endothecium although the tapetum was degenerated (**Figures 2D,F**). Secondary cell wall thickening was necessary to create the shearing force required for anther dehiscence through the stomium (Dawson et al., 1999). At later stages, the septa of L23 anthers were degraded as in the wild type (**Figures 2G,H**). While the stomium broke in L23, the anthers did not open (**Figures 2I,J**). Alexander staining showed that the pollen grains in L23 anthers were viable (Supplementary Figure S2). These observations indicate that non-dehiscence in *p35S:mTCP24* anthers is due to the defect in secondary wall thickening in the anther endothecium.

**FIGURE 3:**
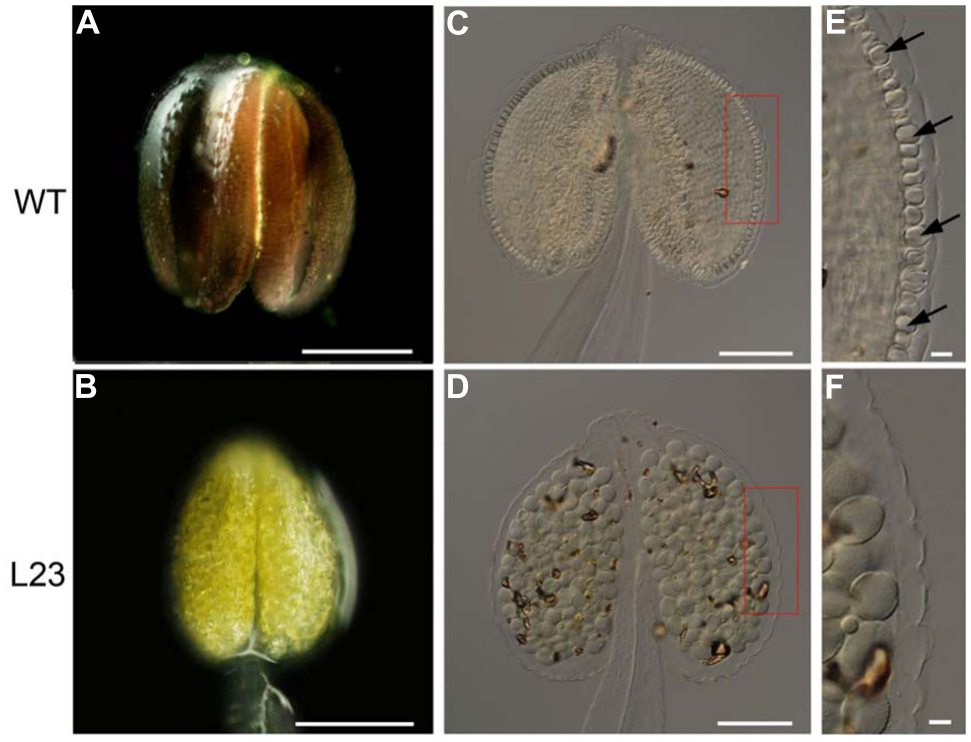
**Secondary cell wall thickening of anthers in the transgenic plants with *p35S:mTCP24*. (A,B)** Phloroglucinol staining of wild type **(A)** and L23 **(B)** anthers. Lignified material was stained in deep red. **(C–F)** Microscopic observation of the anthers treated with clearing fluid. **(C)** The wild type. **(D)** L23 line. **(E,F)** Magnification of red box region in **(C)** and **(D)**. Arrows indicate the thickened endothecium cells. Scale bars: 200 μm in **(A,B)**; 100 μm in **(C,D)**; 10 μm in **(E,F)**.

We examined the accumulation of lignin, which was the major component of secondary walls according to phloroglucinol staining. The deep red staining of lignified materials by phloroglucinol was clearly observed in the endothecium layer in the wild type anthers (**Figure 3A**). However, no staining was observed in L23 anthers (**Figure 3B**). We also treated the anthers with clearing fluid. The thickened cell walls appeared in the wild type endothecium (**Figures 3C,E**) but were absent in the transgenic plants (**Figures 3D,F**). These results indicate that *TCP24* negatively regulates secondary wall thickening in the anther endothecium.

**FIGURE 4:**
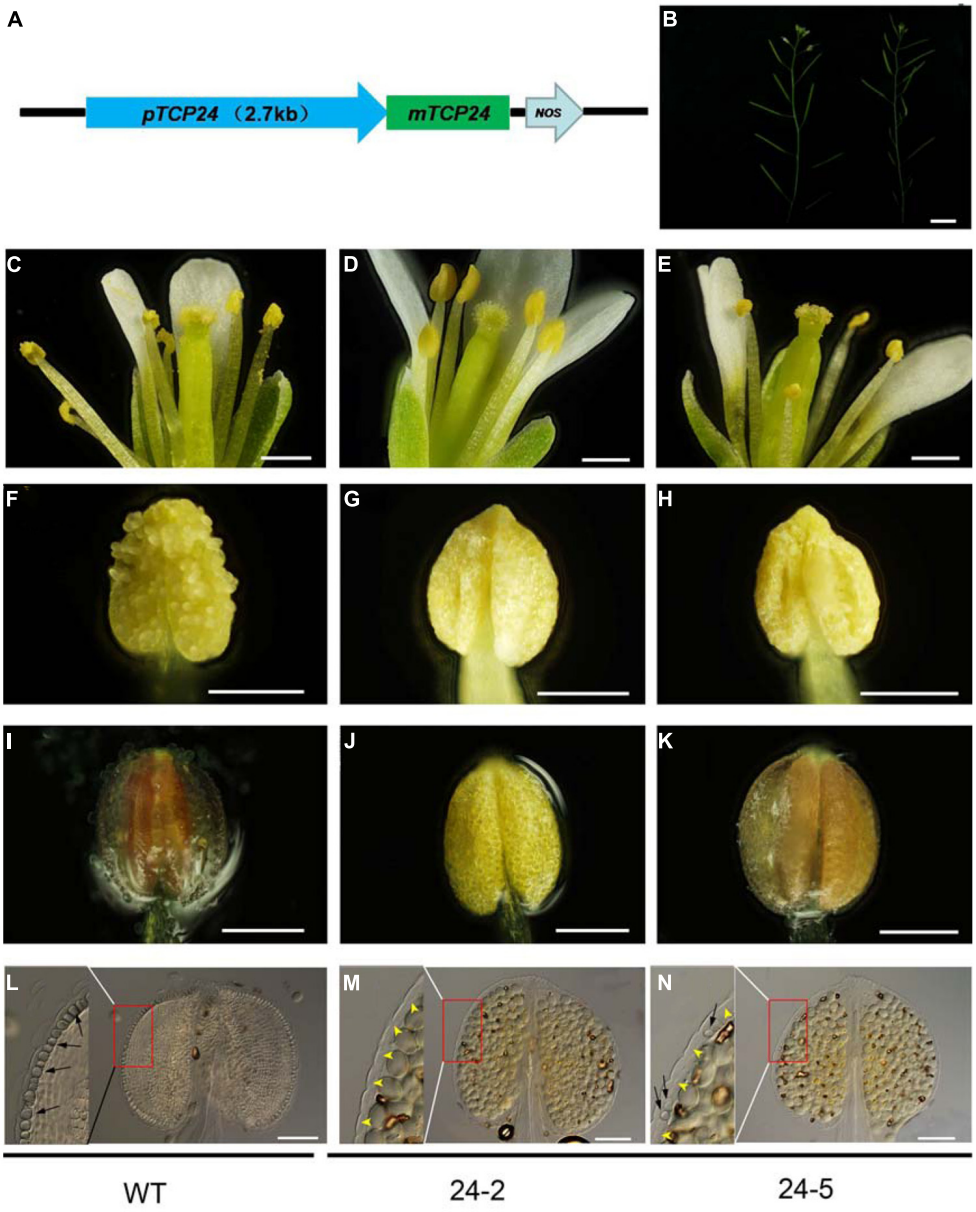
**The floral organs and anthers of the transgenic plants with *pTCP24:mTCP24*. (A)** Diagram of *pTCP24:mTCP24* construct. **(B)** Inflorescences of the wild type (left) and *pTCP24:mTCP24* (24-5; right) plants. **(C–E)** Open flowers in the wild type **(C)**, 24-2 **(D)** and 24-5 **(E)** lines. **(F–H)** Anthers in the wild type **(F)**, 24-2 **(G)**, and 24-5 **(H)** lines. **(I–K)** The wild type **(I)**, 24-2 **(J)**, and 24-5 **(K)** anthers with phloroglucinol staining. **(L–N)** The wild type **(L)**, 24-2 **(M)**, and 24-5 **(N)** anthers treated with clearing fluid. Images in the left of each figures is magnified from the red boxes. Black arrows indicate the thickened endothecium cells, and yellow arrowheads indicate no thickened endothecium cells. Scale bars: 1 cm in **(B)**; 500 μm in **(C–E)**; 200 μm in **(F–K)**; 100 μm in **(L–N)**.

To exclude the effect of the 35S promoter on ectopic expression, we expressed *TCP24* under the control of its native promoter (2.7 kb 5′ upstream of the *TCP24* transcriptional start site) (**Figure 4A**). Among the *pTCP24:mTCP24* lines, some had a complete loss of fertility because seed set was not observed, while most showed a reduced fertility compared with the wild type (**Figure 4B**). In the transgenic line 24-2 which was sterile, there was no pollen on the stigmas, indicating that pollen grain release from the anthers was arrested (**Figures 4C,D**), and no pollen was observed being released from the anthers (**Figures 4F,G**). Under the microscope, a few pollen grains were found to be outside the stomium on 24-5 plants (**Figures 4E,H**). Using phloroglucinol staining, we observed no staining in the anthers of 24-2 plants (**Figure 4J**) and very weak red staining in the anthers of 24-5 plants (**Figures 4I,K**). The cell walls of the endothecium were thickened uniformly in the wild type (**Figure 4L**), but they were not observed in 24-2 anthers (**Figure 4M**). On the transgenic line 24-5 which had reduced fertility, secondary cell walls occurred in some positions (**Figure 4N**). These observations confirmed that the overexpression of *TCP24* inhibited secondary wall thickening in the anther endothecium.

### *TCP24* Gene Expression Became Weak at the Anther Endothecium Initiation Stage

**FIGURE 5:**
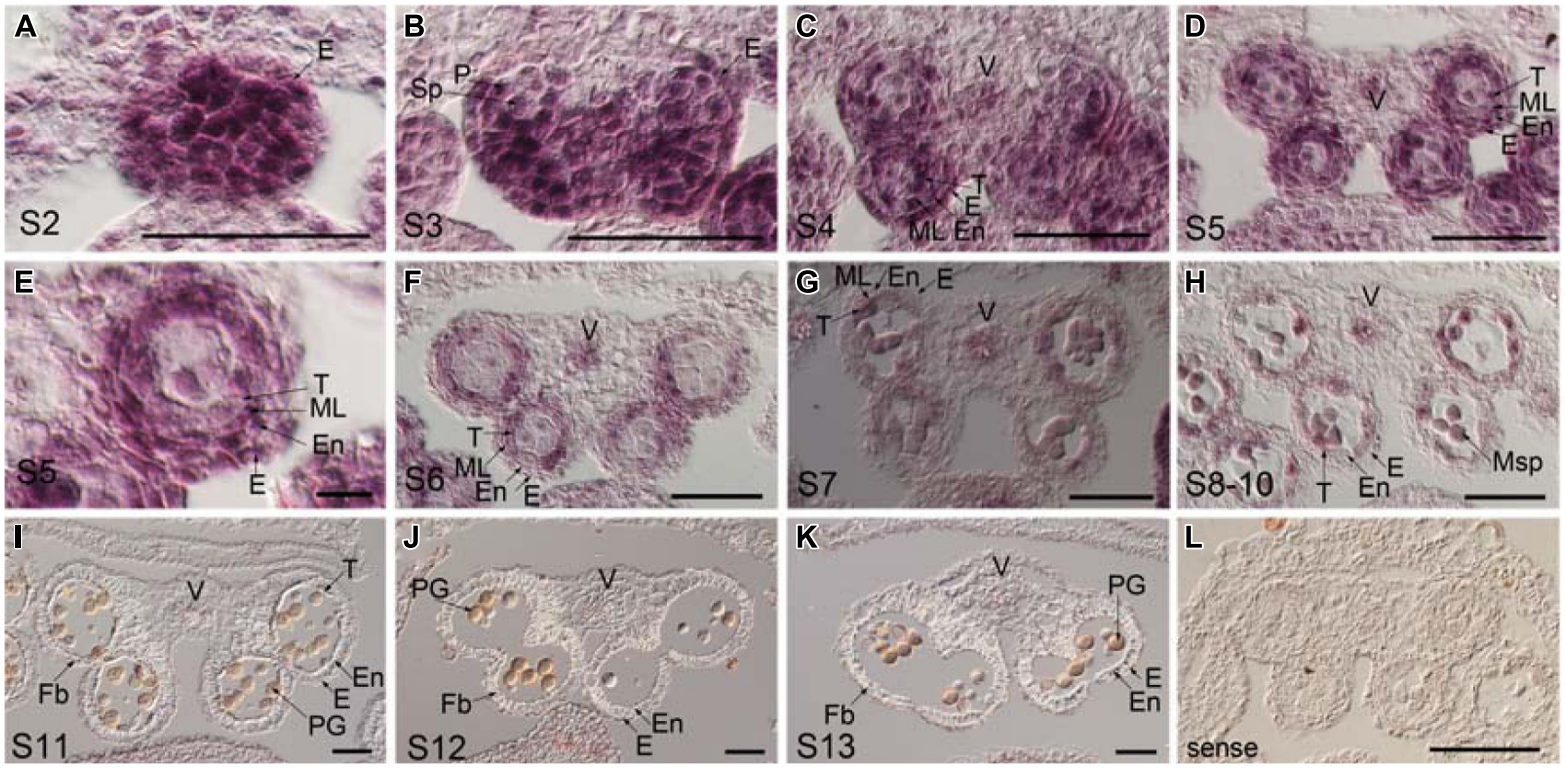
**Temporal and spatial expression patterns of *TCP24* gene during anther development.** *In situ* hybridization using *TCP24* probe. **(A–K)** Transverse sections of anthers at the stages 2 **(A)**, 3 **(B)**, 4 **(C)**, 5 **(D,E)**, **and** 6 **(F)**, 7 **(G)**, 8–10 **(H)**, 11 **(I)**, 12 **(J)**, and 13 **(K)** antisense probes. **(E)** Magnified picture from the top right region in **(D)**. **(L)** Sense probe of *TCP24.* E, epidermis; En, endothecium; Fb, fibrous bands; ML, middle layer; Msp, microspore; P, parietal cell; PG, pollen grain; Sp, sporogenous; T, tapetum; V, vascular region. Scale bars: 50 μm in **(A–D**, **F–L)**; 5 μm in **(E)**.

To examine the temporal and spatial expression of *TCP24* during anther development, *in situ* hybridization was performed using the wild type anthers. The anther development was divided into 14 stages (Sanders et al., 1999). At stage 2, the *TCP24* signal was strong in the whole region (**Figure 5A**). At stage 3, the signal was preferential in the epidermal, parietal layer and sporogenous cells (**Figure 5B**). At stages 4 to 5, when four clearly defined locules were established, *TCP24* was strongly expressed in the epidermis, endothecium, middle layer, tapetum, vascular tissue, and microspore mother cell (**Figures 5C–E**). At stage 6, the signal became weak, and was clearly localized in the tapetum, microspore, and vascular region (**Figures 5F–H**). From stage 11, when secondary cell wall thickening begins, the *TCP24* signal disappeared in the endothecium, but was still present in the vascular region (**Figures 5I–L**). The expression domains of miR319a were similar as those of *TCP24* (Supplementary Figure S3). To address whether *TCP24* overexpression causes the ectopic distribution of *TCP24* in endothecium, we detected *TCP24* in L23 anthers. The *TCP24* expression pattern was the same as that of the wild type at stage 5, although the signal was much stronger than in the wild type (Supplementary Figure S4A). There was no ectopic signal in the endothecium during the secondary wall thickening process (Supplementary Figures S4B–D). These observations suggested that the lack of secondary wall thickening in the anthers of *p35S:mTCP24* plants was due to the high level of *TCP24* rather than its misexpression.

### *TCP24* Regulates the Genes Linked to Secondary Cell Wall Thickening

**FIGURE 6:**
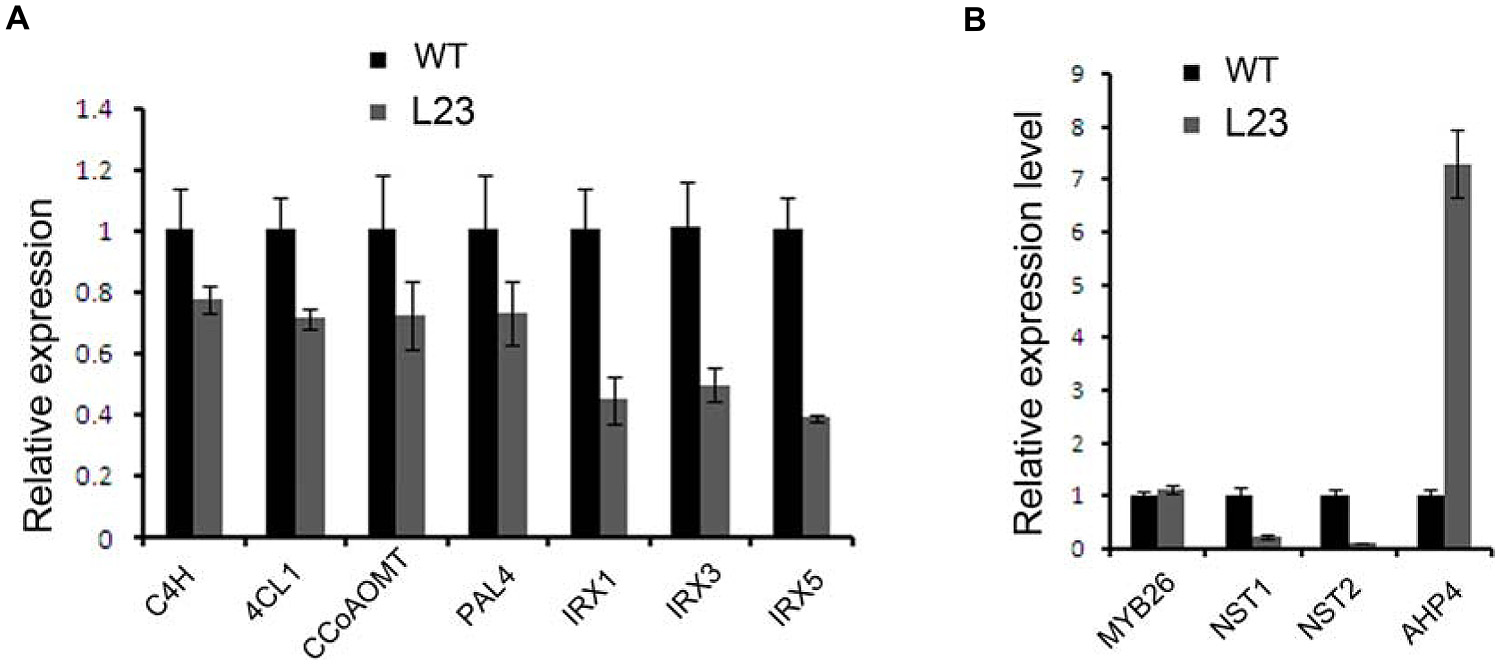
**Regulation of *TCP24* to the genes linked to secondary cell walls. (A)** Relative expression levels of genes involved in the biosynthesis of lignin (*C4H*, *4CL1*, *CCoAOMT*, *PAL4*) and cellulose (*IRX1*, *IRX3*, and *IRX5*). **(B)** Relative expression levels of the genes that regulate the secondary wall thickening. Error bars represent SD of three replicates.

Secondary walls in the anther endothecium are composed of lignin and cellulose. Mutations of the genes involved in these biosynthesis processes cause non-dehiscent anthers (Brown et al., 2005; Thevenin et al., 2011). We examined the expression profiles of the genes involved in the biosynthesis of lignin (*C4H*, *4CL1*, *CCoAOMT*, and *PAL4*) and cellulose (*IRX1*, *IRX3*, and *IRX5*) (Boerjan et al., 2003; Somerville, 2006). All of these genes were down-regulated in the flower buds of *p35S:mTCP24* plants (**Figure 6A**). It was reported that mutations in *MYB26*, *NST1*, and *NST2*, as well as the overexpression of *AHP4*, resulted in the failure of anther dehiscence and that these genes act upstream to regulate secondary wall biosynthesis genes (Dawson et al., 1999; Steiner-Lange et al., 2003; Mitsuda et al., 2005; Yang et al., 2007). We found that *TCP24* overexpression significantly reduced the expression of *NST1* and *NST2*, but not *MYB26*. Unexpectedly, *AHP4* was greatly up-regulated (**Figure 6B**). These results indicate that *TCP24* negatively regulates secondary wall biosynthesis genes and is possibly upstream of these genes in the pathways.

### *TCP24* Silencing is Helpful for Thickening Anther Endothecium Secondary Cell Walls

**FIGURE 7:**
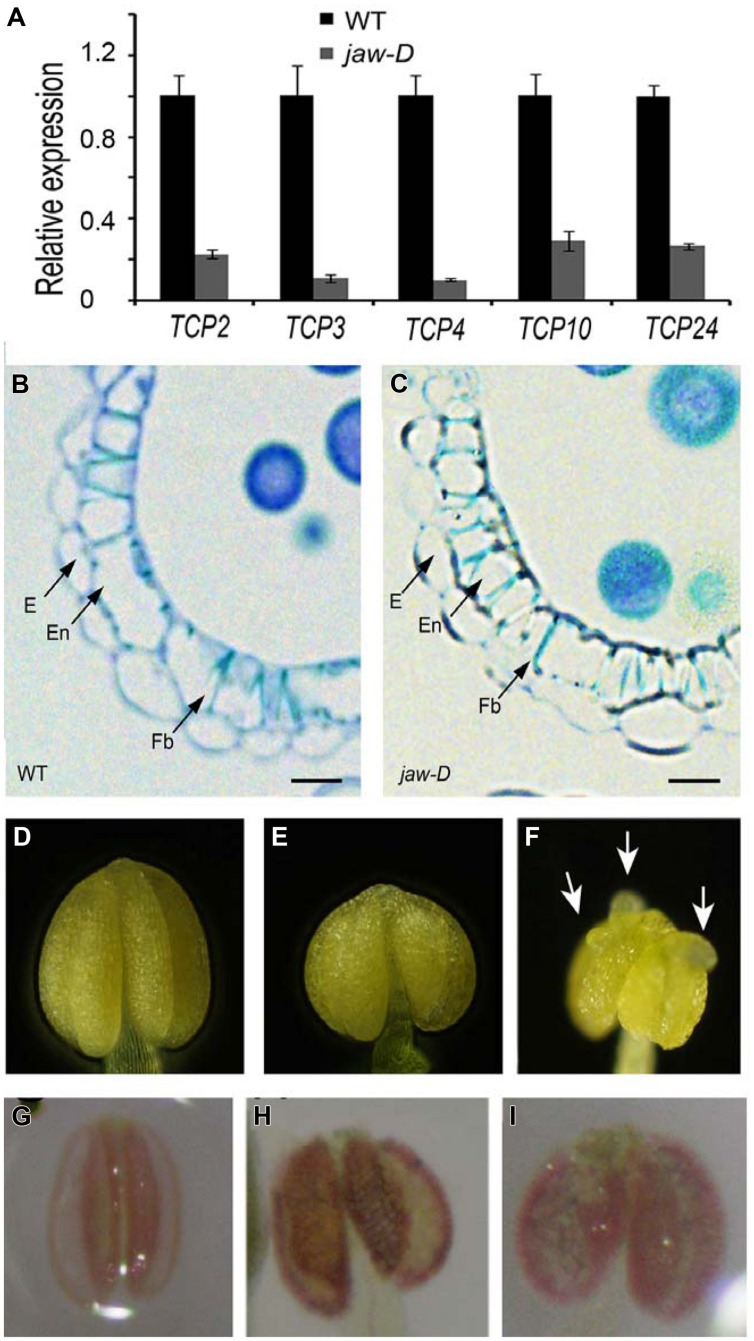
**Secondary cell wall thickening of anther endothecium in the plants with *TCP24* silencing. (A)** Relative expression of miR319a-targeted *TCP* genes in flower buds of *jaw-D* mutants. **(B,C)** Transverse sections of the anthers in the wild type **(B)** and *jaw-D* **(C)** plants. **(D–F)** Anthers of the wild type **(D)** and the *p35S:TCP24SRDX* **(E,F)** plants. Arrows indicate protuberances. **(G–I)** Phloroglucinol staining of anthers showing secondary cell wall thickening of anther endothecium in the wild type **(G)** and the *p35S:TCP24SRDX* **(H,I)** plants (the strong phenotype in the left and weak phenotype in the right). Scale bars: 10 μm in **(B,C)**.

To investigate whether secondary wall thickening was affected by *TCP24*, we carefully observed the anthers of *jaw-D* mutant plants in which miR319a-targeted *TCP* genes were down-regulated (Palatnik et al., 2003). In the flower buds of *jaw-D*, these *TCP* genes were down-regulated (**Figure 7A**). The epidermal tissues and the anther endothecium were particularly affected in the *jaw-D* mutant. The periclinal cell walls were thicker than the wild type (**Figures 7B,C**).

To exclude the redundant effects of the other *TCP* genes, we created *p35S:TCP24SRDX* plants by fusing *TCP24* with the SRDX repression domain. This approach converted transcription factors into dominant repressors, even in the presence of redundant genes (Hiratsu et al., 2003), and has been extensively used to study the functions of *TCP* genes (Koyama et al., 2007, 2010; Guo et al., 2010; Kieffer et al., 2011; Uberti-Manassero et al., 2012). A total of 48 independent transgenic lines were obtained. Their anthers were wider than the wild type (**Figures 7D,E**) and some had protuberances on their surface (**Figure 7F**). Phloroglucinol staining showed that lignification was enhanced and the endothecium layers were much thicker in the anthers of *p35S:TCP24SRDX* plants compared with the wild type (**Figures 7G–I**). This result indicates that the posttranscriptional silencing of TCP24 promotes the thickening of secondary cell walls in the anther endothecium.

**FIGURE 8:**
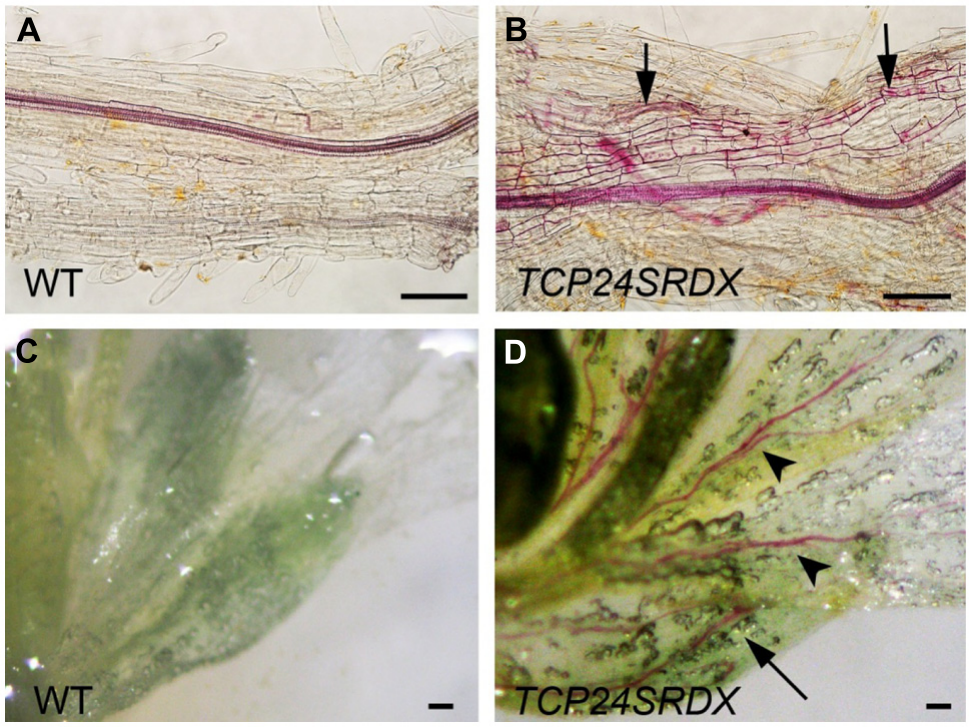
**Ectopic thickening of secondary walls in *p35S:TCP24SRDX* plants.** The tissues were stained with phloroglucinol staining to detect the lignified secondary wall thickening. **(A,B)** Roots of the wild type **(A)** and *p35S:TCP24SRDX* plants **(B)**. Arrows in **(B)** indicate ectopic deposition of secondary walls. **(C,D)** Flowers of the wild type **(C)** and *p35S:TCP24SRDX* plants **(D)**. Arrow and arrowheads in **(D)** indicate enhanced deposition of secondary walls in sepals (arrow) and petals (arrowheads). Scale bars: 100 μm.

Besides the anther endothecium, secondary wall thickening was observed in the other tissues using phloroglucinol staining. In the wild type roots, lignified secondary wall thickening was observed in vascular bundles but not in the parenchymatous cells (**Figure 8A**) as observed (Herve et al., 2009). In *p35S:TCP24SRDX* roots, however, it was seen in the parenchymatous cells as well (**Figure 8B**). In vascular bundles of the mature sepals and petals the transgenic plants exhibited stronger signals of lignified secondary wall thickening compared with the wild type plants (**Figures 8C,D**). These results indicate that *TCP24* repression influences the ectopic thickening of the secondary walls in various tissues.

## Discussion

miR319a-targeted *TCP* genes may play important roles in controlling cell division and differentiation during leaf development (Palatnik et al., 2003; Koyama et al., 2007, 2010; Ori et al., 2007; Efroni et al., 2008; Li et al., 2012b). In this study, we found that miR319a-targeted *TCP24* negatively regulates secondary cell wall thickening in the anther endothecium. This result suggests that miR319a-targeted *TCP* genes are multifunctional in their regulation of cell development. It has also been reported that TCP4 can bind to the *LOX2* promoter, regulating leaf senescence by controlling the expression of jasmonic acid biosynthesis genes (Schommer et al., 2008). The proper level of active *TCP4* is critical for petal and stamen development (Nag et al., 2009), and TCP2 and TCP3 interact with components of the core circadian clock (Giraud et al., 2010). Additionally, TCP3 interacts with R2R3-MYB proteins and participates in the flavonoid biosynthesis pathway (Li and Zachgo, 2013).

In transgenic plants containing *p35S:mTCP24*, secondary cell wall thickening does not occur in the anther endothecium. Overexpression of *TCP24* under its native promoter also exhibits a similar phenotype. However, silencing *TCP24* by enhanced miR319a expression or the repression of TCP24 using the SRDX repressor domain causes increased lignification and the deposition of secondary cell walls in the anther endothecium. Apparently, *TCP24* represses the secondary cell wall thickening in the anther endothecium. In the wild type anthers, *TCP24* strongly expresses in the endothecium when this cell layer is formed, and the expression weakens and eventually disappears when secondary wall thickening occurs. Clearly, *TCP24* acts as a repressor of secondary wall thickening at the early stage of endothecium development.

Secondary wall thickening in the anther endothecium is important for anther dehiscence. Several processes, such as degeneration of the tapetum, septum, and breakage of stomium cells that affect dehiscence, occur normally in the transgenic plants. Pollen grains are fertile but they remain locked into the non-dehiscent anthers. Microscopic observation and histological staining suggest that this defect is due to the lack of secondary wall thickening in the anther endothecium. The importance of this process has been verified in several studies (Dawson et al., 1999; Steiner-Lange et al., 2003; Mitsuda et al., 2005; Yang et al., 2007; Jung et al., 2008; Kim et al., 2012).

A few genes have been linked to secondary thickening in anther endothecium. *MYB26*, *NST1*, and *NST2* positively control secondary thickening by regulating the expression of secondary wall biogenesis genes, and *AHP4* negatively regulates this process (Steiner-Lange et al., 2003; Mitsuda et al., 2005; Yang et al., 2007). Meanwhile, mutations in the genes that encode secondary wall biogenesis, such as *IRX*, *4CL3*, *CCR*, and *CAD*, also result in failed secondary thickening, resulting in the non-dehiscent phenotype (Brown et al., 2005; Gui et al., 2011; Thevenin et al., 2011; Hao et al., 2014). Other mechanisms exist that can be illustrated by studying the function of mitochondrial gamma *CA2* and *CBSX2. p35S:CA2* plants cause a dramatic decrease in the reactive oxygen species production in anthers, which may impair H_2_O_2_-dependent lignin polymerization and deposition in the anther endothecium, resulting in a lack of secondary wall thickening in the endothecium (Villarreal et al., 2009). CBSX2 modulates the H_2_O_2_ status and may be linked to the jasmonic acid response, which in turn controls secondary wall thickening of the anther endothelial cells (Jung et al., 2013). In this study, we demonstrate that several genes linked to secondary wall biogenesis are down-regulated in *TCP24*-overexpressing plants. *NST1* and *NST2* are down-regulated and *AHP4* is up-regulated in the transgenic plants. These results indicate that *TCP24* acts upstream of the genes that promote secondary wall thickening. It has been reported that *MYB26* is an upstream regulator of *NST1* and *NST2* (Yang et al., 2007). However, *MYB26* transcripts are not changed in *TCP24* overexpressing plants. We speculate that *TCP24* functions in a *MYB26*-independent manner.

The deregulation of *TCP24* causes defects not only in the anther endothecium, but also in roots and flower tissues. It has been reported that TCP proteins form homo- and heterodimers, and the latter bind DNA more efficiently than the former (Kosugi and Ohashi, 2002; Danisman et al., 2012). TCP4 can recognize the GGACCA motif, while TCP3 can activate downstream gene expression by directly binding the GGnCCC motif in the respective promoter (Schommer et al., 2008; Koyama et al., 2010). TCP24 may interact with other proteins and bind to the corresponding motifs in the targeted genes to execute its function. Further work is necessary to elucidate the molecular mechanisms of secondary cell wall thickening in plants.

Our results expand the classical roles of *TCP24* in cell division. *TCP24* regulates the genes that encode the enzymes responsible for secondary cell wall biogenesis, which modify cell walls. It will be interesting to determine whether *TCP24* is involved in the relationship between cell division and cell wall development. Further studies on the *TCP* genes will provide insights into gene regulation pathways in cell differentiation.

## Conflict of Interest Statement

The authors declare that the research was conducted in the absence of any commercial or financial relationships that could be construed as a potential conflict of interest.
