# Supplementary material for: TCP24 modulates secondary cell wall thickening and anther endothecium development
Source: Front Plant Sci. 2015 Jun 24;6:436. doi: 10.3389/fpls.2015.00436 (PMC4478849; doi:10.3389/fpls.2015.00436)
Supplement: Supplementary file 1 [file Data_Sheet_1.DOC]

**SUPPLEMENTARY MATERIAL**

**
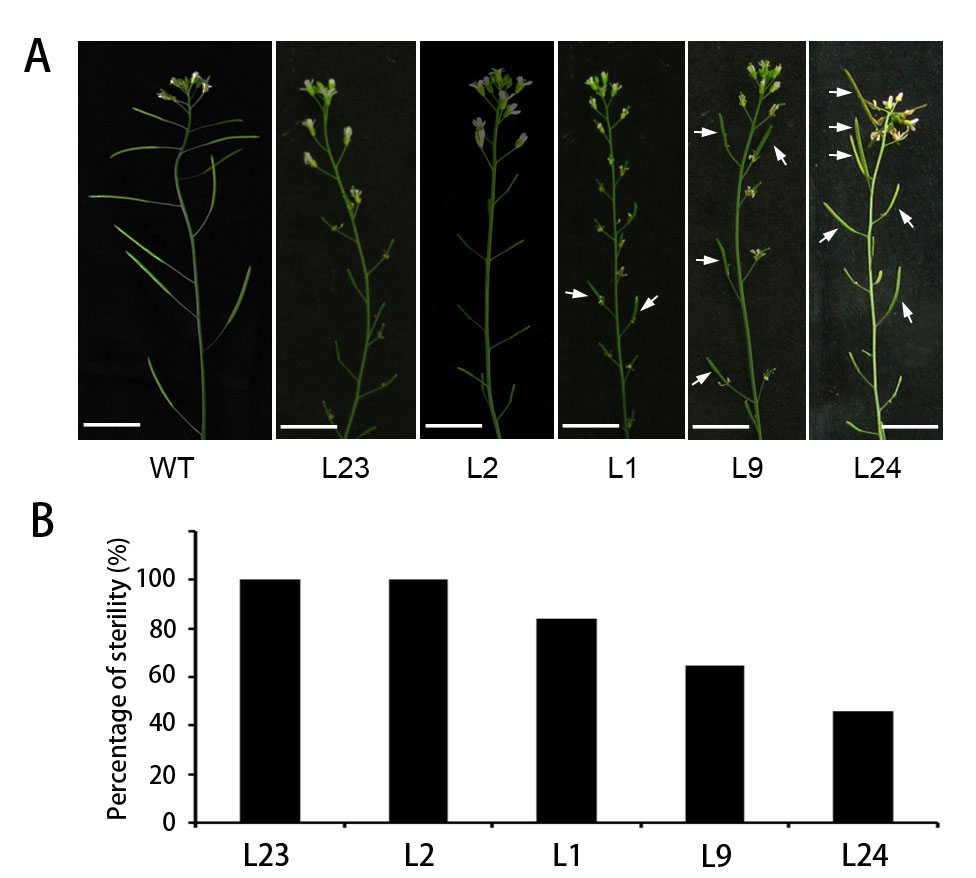
**

**Supplementary Figure S1∣Male sterility in different transgenic lines of *p35S:mTCP24*. (A)** Phenotypes of different transgenic lines of *p35S:mTCP24*. Arrows indicate the fertile siliques. **(B)** Percentage of sterility in different transgenic lines of *p35S:mTCP24*. The numbers of sterile siliques and fertile siliques were counted and the percentage of sterility was calculated. About 100 siliques were counted in each line. Scale bars: 1 cm.

**
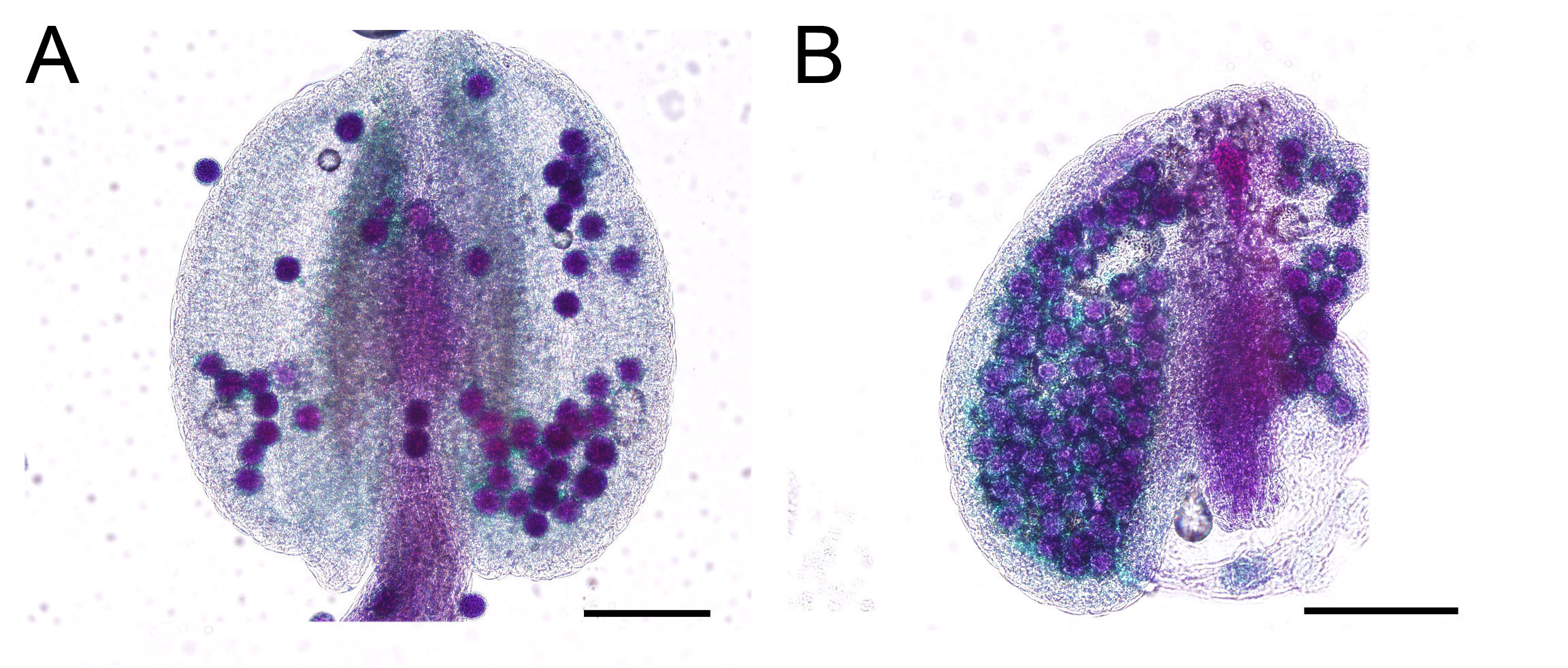
**

**Supplementary Figure S2∣Detection of pollen viability in anthers by** **Alexander staining.** **(A-B)** Anthers of the wild-type **(A)** and *p35S:mTCP24* **(B)** plants at stage 13. Viable pollens are stained in purplish red. Scale bars: 200 μm.


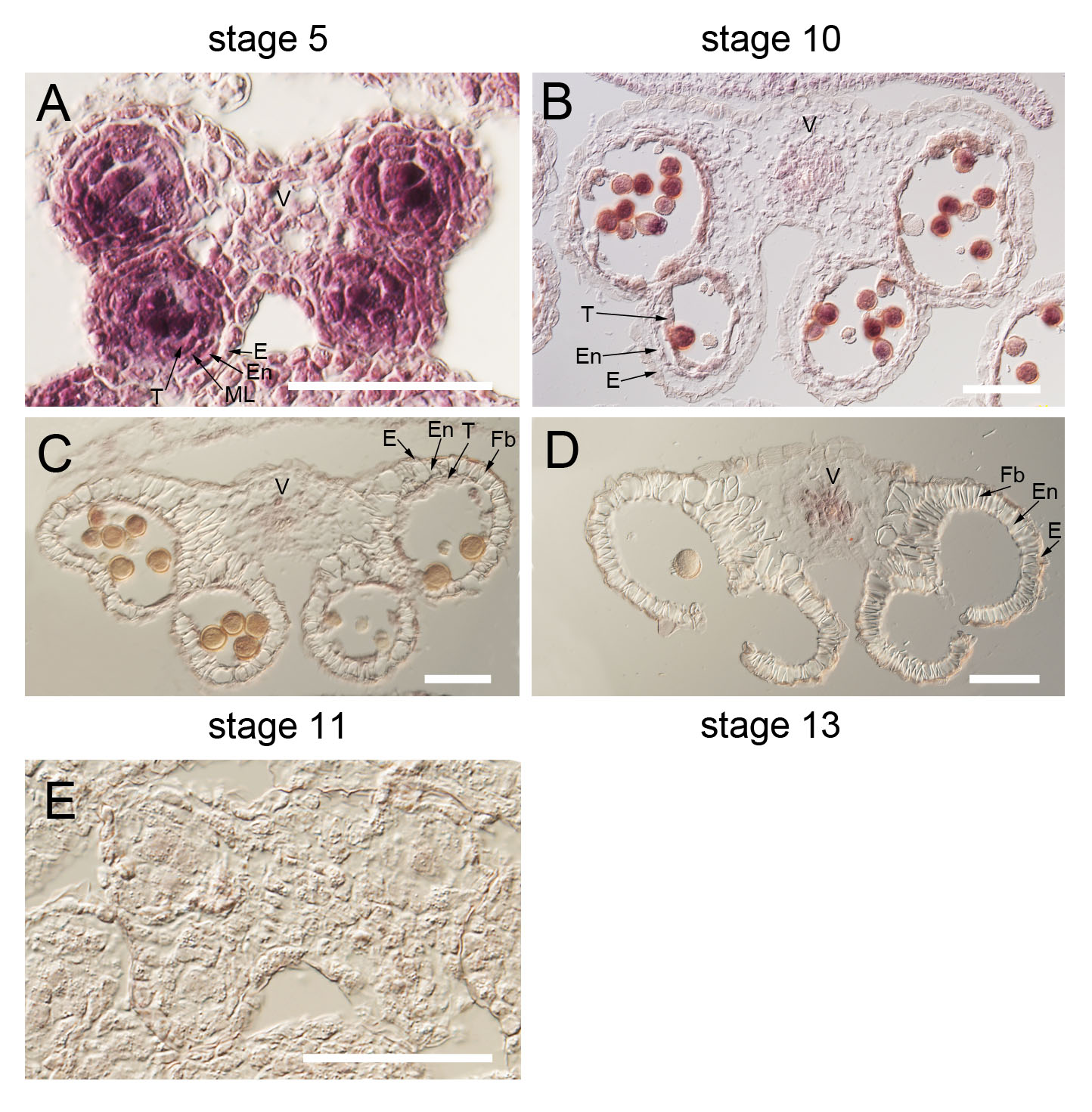


**Supplementary Figure S3∣In situ hybridization analysis of miR319a in the wild-type anthers.** **(A-D)** With antisense probes at stages 5 **(A)**, 10 **(B)**, 11 **(C)** and 13 **(D)**. **(E)** With sense probe at stage 5. E, epidermis; En, endothecium; Fb, fibrous bands; ML, middle layer; T, tapetum; V, vascular region. Scale bars: 50 μm.


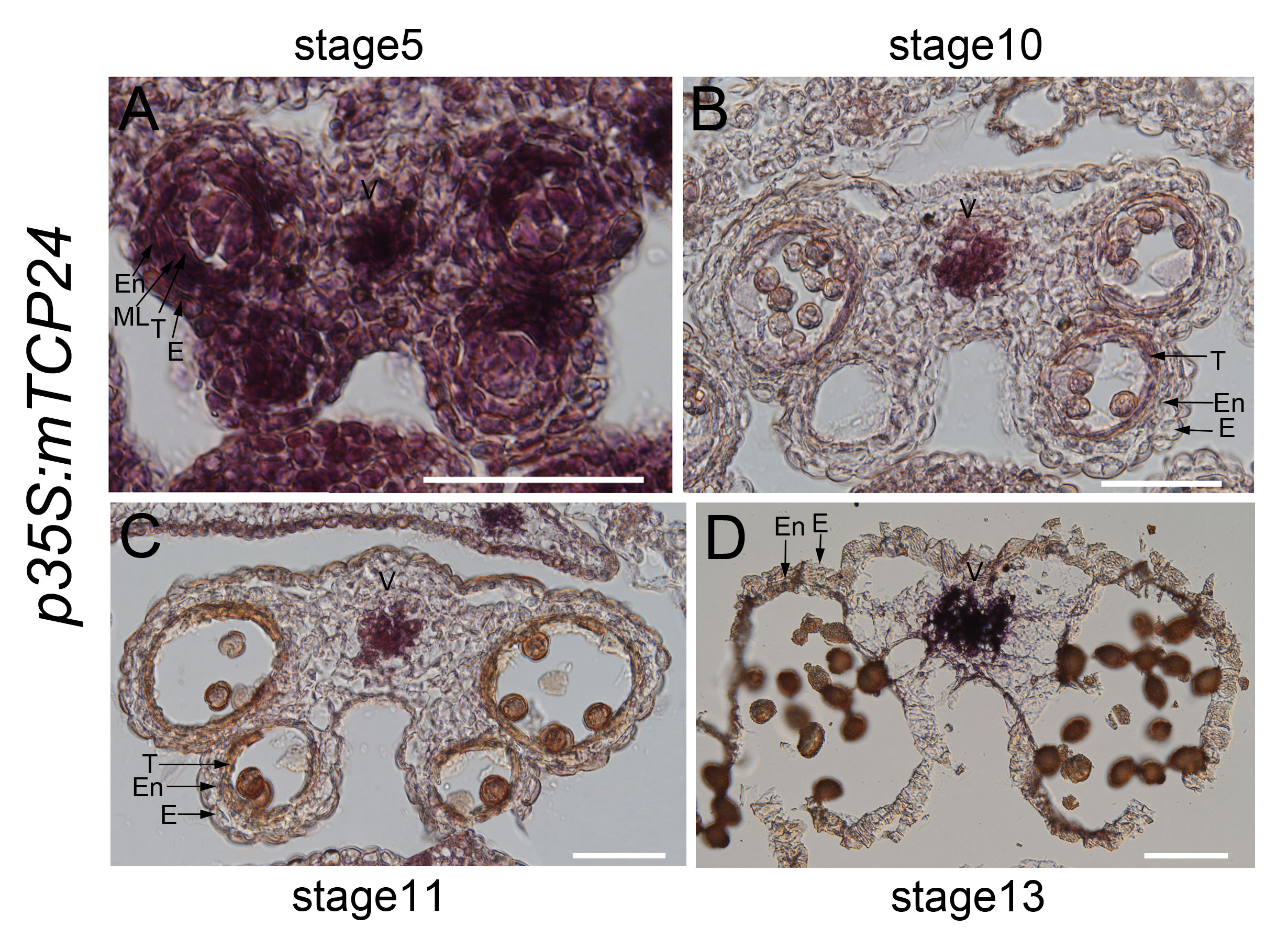


**Supplementary Figure S4∣In situ hybridization analysis of *TCP24* in *p35S:mTCP24* anthers.** **(A)** Stage 5. **(B)** Stage 10. **(C)** Stage 11. **(D)** Stage 13.

E, epidermis; En, endothecium; ML, middle layer; T, tapetum; V, vascular region. Scale bars: 50 um.

**Supplementary Figure S4∣In situ hybridization analysis of *TCP24* in *p35S:mTCP24* anthers.** **(A-D)** With antisense probes at stages 5 **(A)**, 10 **(B)**, 11 **(C)** and 13 **(D)**. E, epidermis; En, endothecium; ML, middle layer; T, tapetum; V, vascular region. Scale bars: 50 um.

**Supplementary Table S1****∣Primers used in this study.**

| Primers for constructs | |
| --- | --- |
| TCP24-S  TCP24-A | ATGGAGGTTGACGAAGACATTG  CTATCTCCTTTCCTTTGCCTTG |
| TCP24-m1  TCP24-m2 | CAGTAGTAGGGGGACCTTGCAAAGTAACTCACAATCTCTCTTTC  GAAAGAGAGATTGTGAGTTACTTTGCAAGGTCCCC  CTACTACTG |
| TCP24 pro-S  TCP24 pro-A | CCGGAATTCCATTTGTGTTATATTCTTAGTTCGG  CGCGGATCCTGTAACGCTACTCTCATACTTTATCAG |
| TCP24-SRDX-S  TCP24-SRDX-A | CGCGGATCCATGGAGGTTGACGAAGACATTG  AACTGCAGATCTCCTTTCCTTTGCCTTG |
| Primers for real time PCR | |
| ACTIN-RT-S  ACTIN-RT-A | TGGCATCAYACTTTCTACAA  CCACCACTDAGCACAATGTT |
| TCP24-RT-S  TCP24-RT-A | GACCAACAACCGAGTAAC  CAAGAGGTGGAGGTGTAG |
| C4H-RT-S  C4H-RT-A | ACTGGCTTCAAGTCGGAGAT  ACACGACGTTTCTCGTTCTG |
| | 4CL1-RT-S  4CL1-RT-A | | --- | | TCAACCCGGTGAGATTTGTA  TCGTCATCGATCAATCCAAT |
| CCoAOMT-RT-S  CCoAOMT-RT-A | CTCAGGGAAGTGACAGCAAA  GTGGCGAGAAGAGAGTAGCC |
| PAL4-RT-S  PAL4-RT-A | GCCGCCGCAATTATGGAACAT  GCTGCTCTGCGCTTTGGACA |
| IRX1-RT-S  IRX1-RT-A | ACGGAGAGTTCTTTGTGGCT  GGTCTGTGTTGGAACAATGG |
| IRX3-RT-S  IRX3-RT-A | CAGGCGTACTCACAAATGCT  TGTCAATGCCATCAAACCTT |
| IRX5-RT-S  IRX5-RT-A | GGATCAGCTCCGATCAATTT  ACCACAAAGGACAATGACGA |
| | MYB26-RT-S | | --- | | MYB26-RT-A | | | CCATGGATGTTGGAGCTCTGTT | | --- | | GCTTCCACGTTTAAGATCAGGTCT | |
| NST1-RT-S  NST1-RT-A | AAGCATCACAAGACGAAGGATG  ATGATGTCGTCTTCGGCGTAT |
| NST2-RT-S  NST2-RT-A | CAATGAGGAGACTATCGAGCAA  TGATAACTCGTGATGGTGGTGT |
| AHP4-RT-S  AHP4-RT-A | CTCCAAGATGATGCAAACCCTAA  TCAGCTTTCACTTTACTTGCCC |
| Probe for in situ hybridization | |
| NA-miR319a | AGGGAGCTCCCTTCAGTCCAA |
